# Concentrated growth factor regulates the macrophage-mediated immune response

**DOI:** 10.1093/rb/rbab049

**Published:** 2021-08-17

**Authors:** Haiyun Luo, Wenjing Liu, Yachuan Zhou, Xiao Jiang, Yeungyeung Liu, Qin Yang, Longquan Shao

**Affiliations:** Department of Endodontics, Stomatological Hospital, Southern Medical University, 366 Jiangnan Avenue South, Guangzhou 510280, China; Department of Prosthodontics, Stomatological Hospital, Southern Medical University, Guangzhou 510280, China; State Key Laboratory of Oral Diseases & National Clinical Research Center for Oral Diseases & Department of Cariology and Endodontics, West China Hospital of Stomatology, Sichuan University, NO. 14, 3rd Section of Ren Min Nan Rd., Chengdu 610041, China; Department of Oral Medicine, Stomatological Hospital, Southern Medical University, Guangzhou 510280, China; Department of Periodontics, Stomatological Hospital, Southern Medical University, Guangzhou 510280, China; Department of Endodontics, Stomatological Hospital, Southern Medical University, 366 Jiangnan Avenue South, Guangzhou 510280, China; Department of Prosthodontics, Stomatological Hospital, Southern Medical University, Guangzhou 510280, China

**Keywords:** concentrated growth factor, immune response, macrophage, signaling pathway

## Abstract

Concentrated growth factor (CGF) is a promising regenerative material that serves as a scaffold and adjunct growth factor for tissue engineering. The host immune response, particularly macrophage activity, plays a critical role in injury repair and tissue regeneration. However, the biological effect of CGF on the immune response is not clear. To enrich the theoretical groundwork for clinical application, the present study examined the immunoregulatory role of CGF in macrophage functional activities *in vitro*. The CGF scaffold appeared as a dense fibrin network with multiple embedded leukocytes and platelets, and it was biocompatible with macrophages. Concentrated bioactive factors in the CGF extract enhanced THP-1 monocyte recruitment and promoted the maturation of suspended monocytes into adherent macrophages. CGF extract also promoted THP-1 macrophage polarization toward the M2 phenotype with upregulated CD163 expression, as detected by cell morphology and surface marker expression. A cytokine antibody array showed that CGF extract exerted a regulatory effect on macrophage functional activities by reducing secretion of the inflammatory factor interleukin-1β while inducing expression of the chemokine regulated on activation, normal T cell expressed and secreted. Mechanistically, the AKT signaling pathway was activated, and an AKT inhibitor partially suppressed the immunomodulatory effect of CGF. Our findings reveal that CGF induces a favorable immune response mediated by macrophages, which represents a promising strategy for functional tissue regeneration.

## Introduction

Platelet concentrate products are biomimetic scaffolds that are rich in growth factors and cytokines and are widely used for wound healing and tissue engineering. Natural fibrin scaffolds provide a microenvironment that is conducive to newly formed tissue ingrowth and contain abundant bioactive molecules that regulate various cell behaviors [1, 2]. With advances in preparation procedures, platelet concentrate products, which started from platelet-rich plasma (PRP) followed by platelet-rich fibrin (PRF), have achieved the latest generation: concentrated growth factor (CGF) [3]. The preparation procedure for CGF is easily conducted in clinical practice by simply centrifugation in a specific and fixed program. Furthermore, CGF does not require other additives, which avoids contamination, toxicity and immune reactions. The modified centrifugation procedure for CGF contributes to a relatively denser fibrin scaffold, which is similar to the natural extracellular matrix (ECM). CGF also contains a higher concentration of growth factors than PRP or PRF, and it exhibits superior regenerative capacity and biomaterial potential [2, 4, 5]. CGF has emerged as a promising biomaterial in tissue remodeling and regeneration, including dermal augmentation and fracture healing, by facilitating epidermal repair, bone formation, nerve repair and angiogenesis. CGF is also widely used in the oral and maxillofacial fields to aid in dental implantations, maxillofacial reconstruction and endodontic regeneration [6–9]. CGF advances tissue regeneration via enhanced endogenous stem cell proliferation and differentiation [10–12]. The success of tissue engineering also depends on pathogen elimination and the establishment of tissue homeostasis by the host immune system [13, 14]. The necessary and critical role of the biomaterial-induced host immune response in tissue remodeling was recognized during the last decade. Cytokines and growth factors derived from autologous platelets and leukocytes modulate host immunity, but the regulatory role of CGF in the immune response is not known.

Tissue remodeling is initiated with blood coagulation, inflammation and host responses to foreign bodies, and it results in newly generated tissue and injury repair, which strongly correlate with immune cell activities [13, 15]. Macrophages are a core innate immune cell type that are involved in the entire regenerative process via modulation of the early inflammatory response, tissue regeneration, healing and scar formation [15]. Upon tissue damage, activated monocytes in the circulating blood infiltrate and migrate to injury sites and mature into macrophages with distinct phenotypes and functions [16]. Macrophages dynamically polarize into classically activated M1 or alternatively activated M2 phenotypes, and this high plasticity plays an essential role in functional regeneration and healing [17]. M1 macrophages secrete large quantities of proinflammatory cytokines, recruit effector cells that defend against pathogens and initiate angiogenesis [18, 19]. M2 macrophages primarily contribute to wound healing and regeneration by producing anti-inflammatory cytokines and certain growth factors. The functional state of macrophages is tightly associated with the local milieu established by bioactive factors and by signaling propagation such as the phosphatidylinositol 3-kinase (PI3K)/protein kinase B (AKT) and Janus kinase (JAK)/signal transducer and activator of transcription (STAT) pathways [20]. Manipulation of the macrophage-mediated immune response is now receiving considerable attention as an important strategy in biotechnical therapies [21]. PRP and derived platelet products exhibit diverse biological effects on macrophage polarization [22, 23], but the effect of CGF on macrophage functional activities and the underlying mechanism have not been explored.

Host immune reactions, particularly macrophage reactions to regenerative materials, are now recognized as important modulators in tissue engineering. Great strides have been made in the mechanisms of CGF advancements in stem cell-based regeneration. However, the potential interaction between CGF and host immunity has not been reported. The present study examined whether and how CGF affected the host immune response *in vitro*. CGF modulated the macrophage-mediated immune response by activating the AKT signaling pathway, which may precede conducive tissue regeneration by preserving a favorable host immune response.

## Methods

### Preparation of the CGF scaffold and extract

The ethics committee of Stomatological Hospital, Southern Medical University approved the entire study (ethical code (2021)04). All subjects were informed of the research purpose, and the study was performed according to the guidelines after written informed consent was obtained. Venous blood was collected from 10 healthy young participants (18–23 years old) in sterile vacuum tubes without anticoagulant additives. Whole blood was immediately centrifuged in a MEDIFUGE CGF medical device (Silfradent, Italy) using a special program as follows: 30 s acceleration, 2 min at 2700 rpm, 4 min at 2400 rpm, 4 min at 2700 rpm, 3 min at 3000 rpm and 36 s deceleration [24]. The CGF preparation procedure is different from the basic two-step centrifugation process used to prepare PRP and the single centrifugation process used to obtain PRF at a fixed speed of 3000 rpm for 10 min [3]. The middle buffy coat layer of whole blood was isolated to obtain CGF gel, and specialized plier for membrane creation (Silfradent, Italy) was used to squeeze out the fluids. CGF-conditioned medium (CCM) was prepared as described in a previous study, with slight modifications for the *in vitro* study [25]. Every two CGF gels were mixed into one group before freezing overnight in a vacuum freeze dryer and were then immersed in 20 ml of blank Dulbecco’s modified Eagle’s medium (DMEM; HyClone, USA) at 37°C for 48 h to harvest cytokines [24]. The obtained medium was filtered through a 0.22-µm filter and defined as 100% CCM. CCM (100%) was diluted with DMEM to 50%, 20%, and 10% for use in subsequent studies.

### Characterization of the CGF scaffold

CGF scaffolds were carefully cut into small pieces, and THP-1 macrophages were seeded on the scaffolds. Scaffolds and macrophage-seeded scaffolds were fixed with a 2.5% glutaraldehyde solution and further dehydrated in a gradient of ethanol solutions. The samples were sputter-coated, and scanning electron microscopy (SEM) images were captured to assess ultrastructural features. Representative images were acquired using a scanning electron microscope (HITACHI S-3400N) operating at an accelerating voltage of 15 kV and a working distance of 8–10 mm.

For histological analyses, CGF fibrin scaffolds were fixed with 4% paraformaldehyde, dehydrated and embedded in paraffin. The samples were then sectioned for hematoxylin–eosin staining using a standard protocol.

### Monocyte migration assay

Human monocyte lineage THP-1 cells were pretreated with serum-free Roswell Park Memorial Institute 1640 medium for 24 h, and 200 μL of cells suspended at a density of 2 × 10^6^ cells/ml was added to the top chamber. CCM (600 μL) at concentrations of 0%, 10%, 20% or 50% was placed in the bottom chamber of a Transwell chamber plate with an 8-μm pore size (3464, Corning, USA). Non-migrated cells on the upper membrane were removed after 6 h, and migrated cells attached to the lower membrane were fixed and stained with crystal violet (C0121, Beyotime Biotechnology, China). Microscopic fields were screened, and adherent cell numbers were calculated using ImageJ software.

### Cell adhesion assay

THP-1 monocytes (2 × 10^5^ cells/well) were cultured with CCM at different concentrations for 48 h in 96-well plates. THP-1 monocytes (2 × 10^4^ cells/well) were cultured with CCM and PMA phorbol 12-myristate 13-acetate (PMA, P1585, 100 ng/ml, Sigma, USA) for 48 h [26]. Non-adherent cells were removed with PBS, and the remaining adherent cells were fixed and stained with crystal violet. Five independent microscopic fields were screened and analyzed using ImageJ software.

### Flow cytometry analysis of surface markers

THP-1 monocytes were pretreated with PMA for 24 h to become THP-1 macrophages. THP-1 macrophages cultured in CCM were prepared as single-cell suspensions of 1 × 10^6^ cells/ml and then incubated with the following primary antibodies: BB515-anti-CD80 (1:20, 565009, BD Biosciences, USA), BV421-anti-CD86 (1:20, 562433, BD Biosciences, USA), PE-anti-CD163 (1:20, 560933, BD Biosciences, USA) and APC-anti-CD206 (1:20, 561763, BD Biosciences, USA) [27]. After washing with PBS containing 2% FBS, the cells were analyzed using a FACScan flow cytometer (Becton Dickinson, USA) according to the manufacturer’s protocol.

### Immunofluorescence staining

THP-1 macrophages were cultured in CCM with or without AKT inhibitor (1 μM, AKT inhibitor IV, sc-203809, Santa Cruz, USA) for 48 h. The cells were fixed with paraformaldehyde, permeabilized and subsequently immunolabeled with an antibody against CD163 (1:100, bs-2527R, Bioss Antibody, China). After washing with PBS, THP-1 macrophages were incubated with a FITC-conjugated secondary antibody (1:200, SA00003-2, Proteintech Group, China) and counterstained with 4′,6-diamidino-2-phenylindole. The samples were screened, and images of independent fields were obtained using an automatic fluorescence microscope (Olympus Corporation, USA).

### Polymerase chain reaction

Total RNA was extracted from CCM-treated THP-1 macrophages using RNeasy Mini kit (RE-03113, Foregene Biotec, China) and then subjected to reverse transcription using a commercial kit (RR036A, Takara Biotechnology, Japan). Quantitative polymerase chain reaction (qPCR) was performed using TB Green Fast qPCR Mix (RR430A, Takara Biotechnology, Japan) according to the manufacturer’s protocol. Relative mRNA expression was analyzed using the standard curve method and normalized to the glyceraldehyde-3-phosphate dehydrogenase (GAPDH) mRNA level. The following primers were used in this study: GAPDH, F, 5′-TCAACAGCGACACCCACTC-3′ and R, 5′-GCTGTAGCCAAATTCGTTGTC-3′; interleukin (IL)-1β, F,5′-AAGGCGGCCAGGATATAACT-3′ and R, 5′-TACGGCCTAAGGCAGGCAGTTG-3′; IL-7, F, 5′-TTTTATTCCGTGCTGCTCGC-3′ and R, 5′-AGTGTTCTTTAGTGCCCATCAAAAT-3′; regulated on activation, normal T cell expressed and secreted (RANTES), F, 5′-CAGTCGTCCACAGGTCAAGG-3′ and R, 5′-TCTTCTCTGGGTTGGCACAC-3′; and macrophage chemoattractant protein (MCP)-1, F, 5′-CTGCTCATAGCAGCCACCTT-3′ and R, 5′-CAGGTGACTGGGGCATTGAT-3′.

### Western blot analysis

THP-1 macrophages were cultured in CCM with or without an AKT inhibitor before lysis in a buffer containing a mixture of proteinase and phosphatase inhibitors (P1045, Beyotime Biotechnology, China). Protein samples (10–30 μg) from each group were subjected to SDS-polyacrylamide gel electrophoresis and then transferred to a membrane. The transferred proteins were gently reacted with primary antibodies overnight at 4°C, washed three times and incubated with a secondary antibody for 1 h at room temperature. Primary antibodies against the following proteins were used in this study: PI3K (1:1000; sc-1637, Santa Cruz, USA), phosphorylated AKT (1:1000; 66444-1-Ig, Proteintech Group, China), AKT (1:1000; sc-55523, Santa Cruz, USA), phosphorylated JAK (1:1000; 3771, Cell Signaling Technology, USA), JAK (1:1000; sc-390539, Santa Cruz, USA), phosphorylated STAT3 (1:1000; sc-8059, Santa Cruz, USA), STAT3 (1:1000; 10253-2-AP, Proteintech Group, China) and GAPDH (1:5000; 60004-1-Ig, Proteintech Group, China). Proteins were visualized with signal-enhanced chemiluminescence reagents.

### Antibody arrays

THP-1 macrophages were cultured with 0, 10, 20 or 50% CCM for 48 h, and the supernatants were assayed using Human Inflammation Antibody Array C3 (AAH-INF-3-8, RayBiotech, USA) according to the manufacturer’s protocol. A Gene Ontology (GO) enrichment analysis of differentially secreted cytokines was performed.

### Enzyme-linked immunosorbent assay

The supernatants of THP-1 macrophages cultured in CCM with or without the AKT inhibitor for 48 h were collected after centrifugation. The supernatants were carefully subjected to Enzyme-linked immunosorbent assays (ELISAs) for RANTES (KE00093, Proteintech Group, China) and IL-1β (KE00021, Proteintech Group, China) using a standard protocol.

### Statistical analysis

All experiments were performed at least three times, and values are presented as the mean ± standard deviation. One-way analysis of variance and corresponding *post hoc* tests were used to evaluate the significance of differences. Differences with a *P* values < 0.05 were considered statistically significant and were analyzed using GraphPad PRISM 7.0 software.

## Results

### Morphological features and cytocompatibility of the CGF scaffold

**Figure 1.**
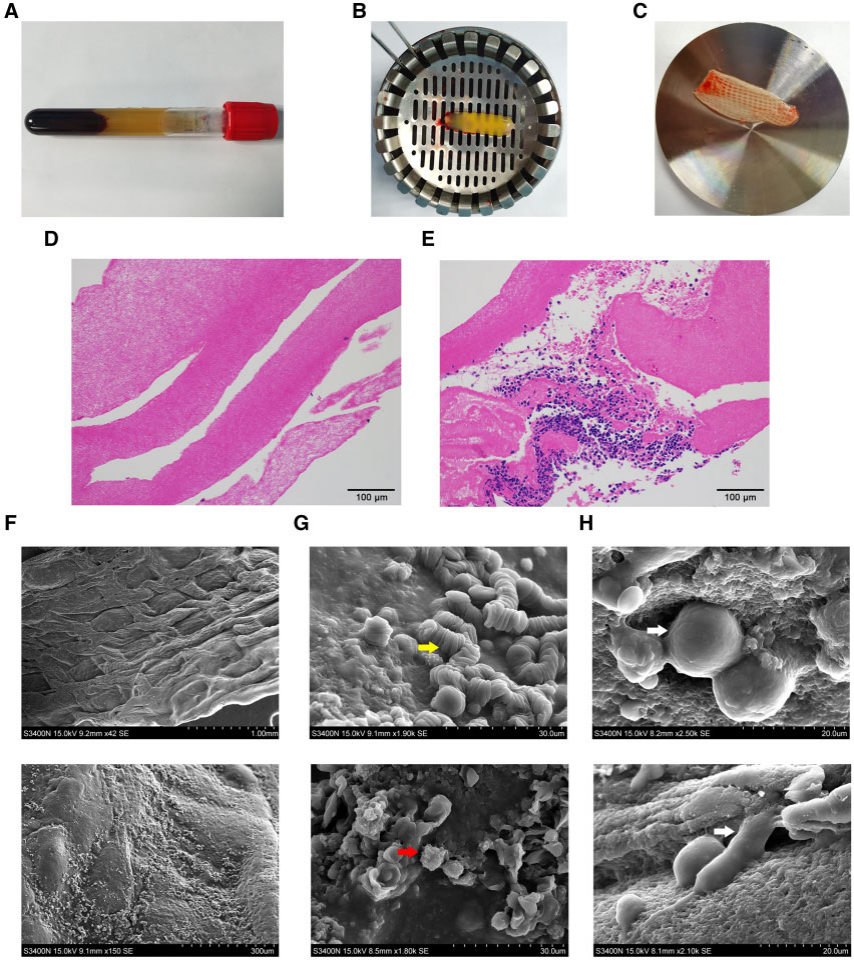
Morphological features and cytocompatibility of the CGF scaffold. (A) Ten milliliters of whole blood after centrifugation. (B) A buffy coat of CGF gel separated from the blood sample. (C) The fibrin network membrane of the CGF scaffold after compression. (D) Histological features of the upper part of the CGF scaffolds evaluated using H & E staining. (E) Leukocytes and red blood cells embedded in the lower portion. (F) The surface ultrastructure of the CGF scaffold observed by scanning electron microscopy. (G) Platelets (yellow arrow) and leukocytes (red arrow) embedded in CGF scaffolds. (H) Macrophages (white arrow) seeded on the CGF scaffolds were fully stretched on the network surface.

A buffy coat of CGF gel was obtained from whole blood using specific centrifugal procedures (Fig. 1A and B). The gel was mechanically compressed into a CGF scaffold that appeared as a tough fibrin network membrane (Fig. 1C). The histological study revealed a fibrin network of the CGF scaffold with leukocytes and red blood cells embedded in the lower portion (Fig. 1D and E). The surface ultrastructure of the CGF scaffold exhibited a dense and gridded texture containing multiple red blood cells, platelets and leukocytes (Fig. 1F and G). THP-1 macrophages seeded onto the CGF scaffold were fully spread on the network surface, as detected by SEM (Fig. 1H).

### CGF extract induced monocyte migration and maturation

**Figure 2.**
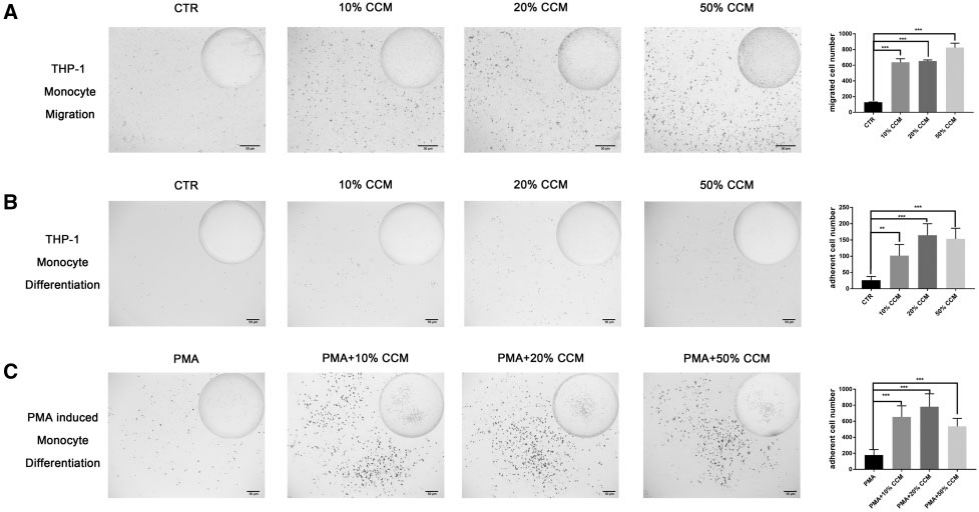
CGF extract induced monocyte migration and maturation. (A) THP-1 monocyte migration increased significantly in the presence of CGF-conditioned medium (CCM), as determined by a Transwell assay (*n* = 3). (B) CCM significantly induced fusion and adherence of THP-1 monocytes (*n* = 5). (C) The number of adherent PMA-induced THP-1 macrophages was significantly increased with CCM treatment (*n* = 5). The data are presented as the mean ± SD. Significance was determined using one-way analysis of variance (ANOVA) and *post hoc* Dunnett tests: *P <* *0.05, **P <* *0.01 and ***P <* *0.001. CGF-conditioned medium (100% CCM) was diluted with medium to 50, 20 and 10% CCM, and medium lacking CCM served as the control (CTR).

The capacity of CGF to recruit monocytes/macrophages was evaluated using Transwell migration assay. The number of migrated THP-1 cells in response to CCM at different concentrations was nearly four times higher than that in the control group, which indicated that CGF promoted monocyte migration (Fig. 2A).

The maturation of monocytes into macrophages was assessed via the transformation of non-adherent monocytes into adherent macrophages. CCM treatment promoted non-adherent THP-1 monocyte differentiation into adherent macrophages (Fig. 2B). PMA was a powerful promoting agent of THP-1 monocyte maturation, and CCM further enhanced the facilitating effects of PMA (Fig. 2C). The adherent cell number in the CCM group was increased 3-fold compared with the control group, and no significant difference was observed between the different concentrations of CCM (Fig. 2B and C).

### CGF extract promoted macrophage polarization toward the M2 phenotype

**Figure 3.**
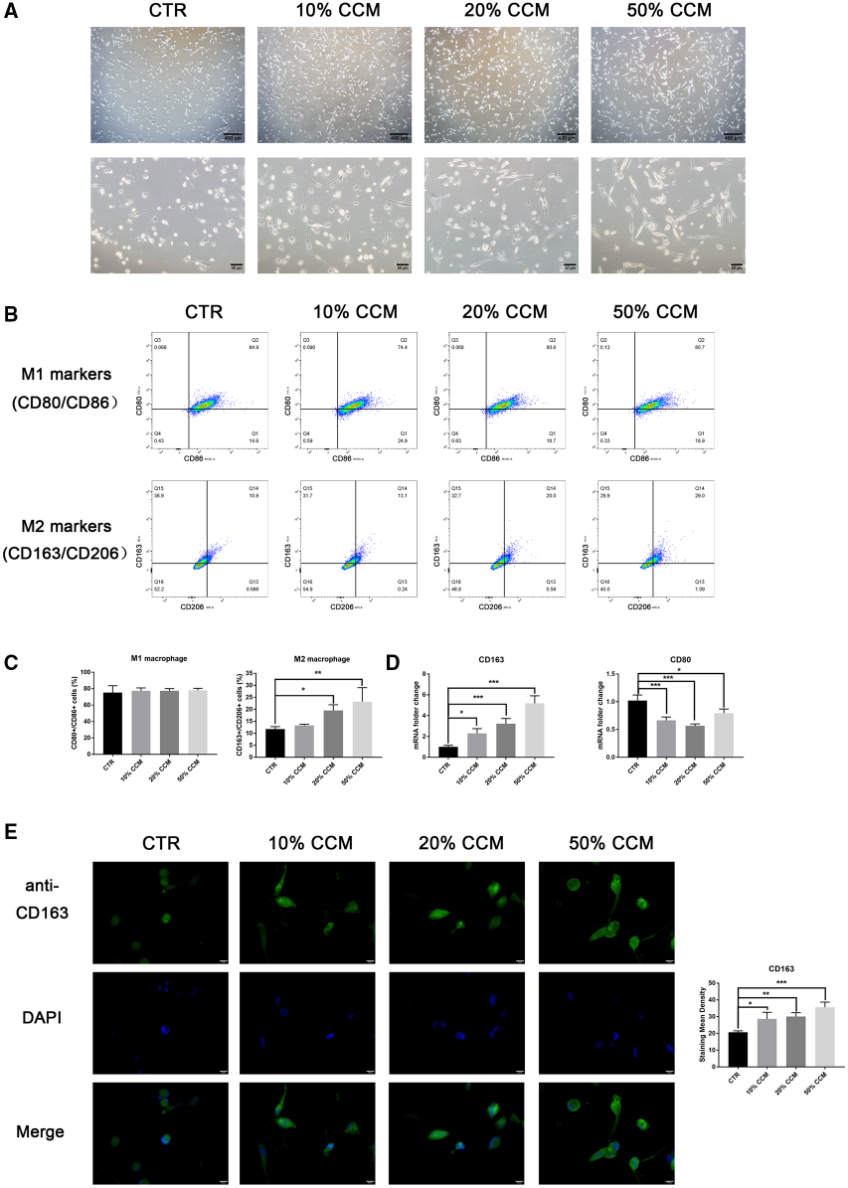
CGF extract promoted macrophage polarization toward the M2 phenotype. (A) Morphological features of CCM-treated THP-1 macrophages were observed under an inverted microscope. CCM groups showed a smaller nuclear-to-cytoplasmic ratio and an elongated cell shape. (B) The M2 (CD163+/CD206+) and M1 (CD80+/CD86+) macrophage phenotypes were analyzed using flow cytometry. (C) The percentage of M2 macrophages increased after CCM treatment, but no variation was observed in the percentage of M1 macrophages. (D) The expression of CD163 mRNA was upregulated, and CD80 mRNA was downregulated in THP-1 macrophages cultured with CCM. (E) Green fluorescent staining of CD163 was increased in THP-1 macrophages treated with CCM. DAPI staining of the nucleus is shown in blue. The quantitative data are presented as the mean ± SD (*n* = 3), and significance was determined using one-way analysis of variance (ANOVA) and *post hoc* Dunnett tests: *P <* *0.05, **P <* *0.01 and ***P <* *0.001.

CCM-treated THP-1 macrophages exhibited a smaller nuclear-to-cytoplasmic area ratio, more granules and a more elongated shape than control macrophages, which are known characteristic morphological features of M2-like macrophages (Fig. 3A, supplementary S1). We investigated the effect of CGF on macrophage polarization via the identification of M1 macrophages (using CD80 and CD86 as markers) and M2 macrophages (using CD163 and CD206 as markers) using flow cytometry. The percentage of CD163+/CD206+ cells increased approximately 1.5 times with the 20 and 50% CCM treatments, but no change was found in the percentage of CD80+/CD86+ cells (Fig. 3B and C). These findings also support the hypothesis that CGF promotes macrophage polarization toward M2-like macrophages. The mRNA expression of the M2 marker CD163 was significantly upregulated with increasing CCM concentrations, but the M1 marker CD80 was downregulated upon CCM treatment (Fig. 3D). Furthermore, immunofluorescent staining for cytoplasmic CD163 gradually increased 1.5-fold with CCM treatment, which indicates that CGF facilitates macrophage polarization (Fig. 3E).

### CGF extract modulated macrophage cytokine secretion

**Figure 4.**
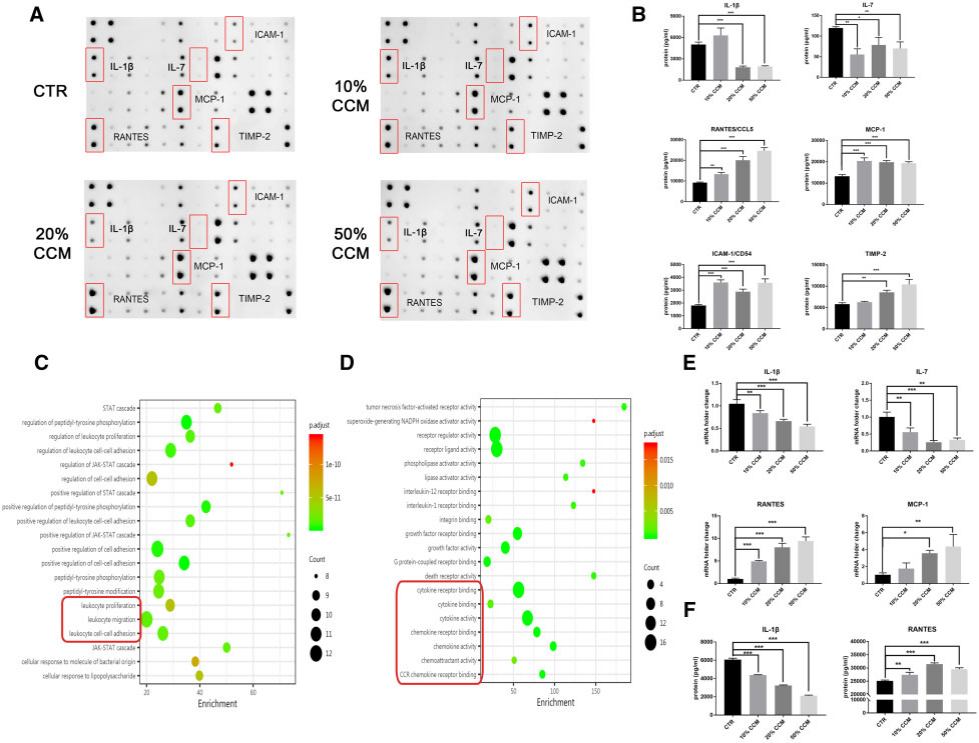
CGF extract modulated macrophage cytokine secretion. (A) The supernatants of CCM-treated macrophages were screened using a human cytokine antibody array. (B) Representative cytokines are presented. (C) A GO analysis of differentially expressed cytokines enriched in BP terms after 20% CCM treatment. (D) Enriched MF categories. (E) The mRNA expression of differentially expressed cytokines (IL-1β, IL-7, RANTES and MCP-1) in CCM-treated macrophages was analyzed using q-PCR. (F) The secretion of IL-1β and RANTES by CCM-treated macrophages was further confirmed using ELISAs. The quantitative data are presented as the mean ± SD (*n* = 3) and were analyzed using one-way analysis of variance (ANOVA) and *post hoc* Dunnett tests: *P <* *0.05, **P <* *0.01 and ***P <* *0.001.

To explore the immunoregulatory effect of CGF on macrophage activation, the secretion of 42 crucial cytokines related to inflammation and immunity was assessed using a human cytokine antibody array (Fig. 4A). Levels of the inflammatory cytokines IL-1β and IL-7 were reduced in the supernatants of THP-1 macrophages treated with CCM, whereas the levels of the chemokines RANTES and MCP-1 were increased. For the ECM, intercellular adhesion molecule-1 expression was upregulated, and tissue inhibitor of metalloproteases (TIMP)-2 expression was downregulated, which might protect the ECM from degradation (Fig. 4B). GO analysis was performed to enrich differentially secreted cytokines in the biological process (BP) and molecular function (MF) categories. At a concentration of 20% CCM, CGF extract modulated macrophage cytokine secretion related to BP terms such as leukocyte migration, proliferation and cell–cell adhesion (Fig. 4C), and the enriched MF terms were receptor activity and cytokine and chemokine activity (Fig. 4D).

We investigated the mRNA expression of representative cytokines, and IL-1β, IL-7, RANTES and MCP-1 mRNA expression levels were consistent with the results from the cytokine antibody array (Fig. 4E). In addition, the ELISA results also confirmed that IL-1β secretion was reduced by half, and RANTES secretion was significantly induced after CCM treatment (Fig. 4F). These data indicate that CGF has a functional effect on the immune response by suppressing the inflammatory cytokine secretion and enhancing chemokine production in immune cells.

### CGF extract regulated the macrophage-mediated immune response via the AKT pathway

**Figure 5.**
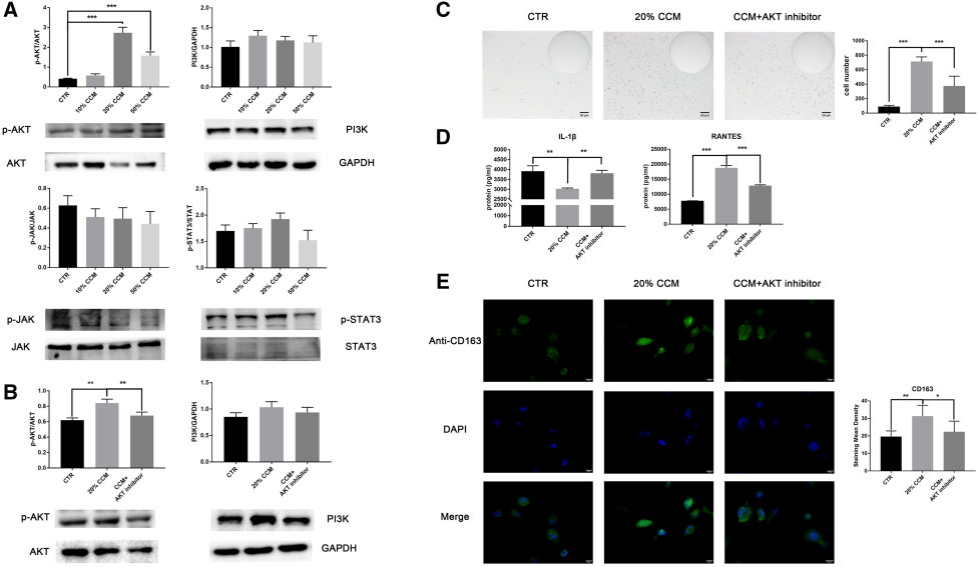
CGF extract regulated the macrophage-mediated immune response via the AKT pathway. (A) A western blot analysis of the proteins involved in the PI3K/AKT (p-AKT, AKT and PI3K) and JAK/STAT (p-JAK, JAK, p-STAT3 and STAT3) signaling pathways in CCM-treated THP-1 macrophages. AKT phosphorylation was increased, but no significant differences were observed in the JAK/STAT pathway (*n* = 3). (B) The effect of an AKT inhibitor on AKT pathway inhibition was analyzed using western blotting (*n* = 3). (C) Representative images of adherent THP-1 macrophages cultured in CCM with or without AKT inhibitor were analyzed using ImageJ software (*n* = 5). (D) The secretion of IL-1β and RANTES by THP-1 macrophages were assayed using ELISAs (*n* = 3). (E) Expression of the M2 subtype marker CD163 was detected using immunofluorescence staining (*n* = 3). The data are presented as the mean ± SD and were analyzed using one-way analysis of variance (ANOVA) and *post hoc* tests: *P <* *0.05, **P <* *0.01 and ***P <* *0.001.

We examined the possible mechanisms involved in the immunoregulatory effect of CGF, and activation of the PI3K/AKT and JAK/STAT signaling pathways was analyzed. The level of phosphorylated AKT was significantly increased 3–5-fold in the 20 and 50% CCM groups, but no trend was observed for the JAK2/STAT3 pathway, which indicates that the AKT pathway was activated in CGF-mediated signaling dynamics (Fig. 5A). An AKT inhibitor was used to inhibit CCM-induced AKT phosphorylation (Fig. 5B). We found that the AKT inhibitor partially suppressed the promoting effect of CCM on monocyte differentiation (Fig. 5C). IL-1β and RANTES secretion and CD163 expression were also reversed by the AKT inhibitor, which indicates that the AKT inhibitor partially abrogated the adjunct effect of CCM (Fig. 5D and E). These data provide evidence that AKT signaling activation contributes to the regulatory role of CGF in macrophage immunoactivity.

## Discussion

As the latest generation of autologous platelet concentrate products, CGF is increasingly used as a regenerative biomaterial in wound healing and tissue regeneration. CGF is a well-recognized activator of endogenous stem cells, but the present study demonstrated an immunoregulatory role of CGF in the macrophage-mediated immune response and its underlying mechanism. CGF induced THP-1 monocyte/macrophage transition, promoted macrophage polarization and modulated cytokine secretion *in vitro*, which correlated with the AKT pathway activation. The present study indicates that CGF elicits a favorable immune microenvironment mediated via macrophage regulation, which might be conducive to tissue regeneration.

CGF is characterized as a relatively tough cross-linked fibrin scaffold containing abundant growth factors and cytokines. The present study characterized the morphological features of CGF scaffolds and the immunoregulatory effect of bioactive molecules in CGF extracts to provide an entry point for exploring the potential role of CGF in the innate immune response. Human monocyte lineage THP-1 cells are very similar to peripheral blood mononuclear cells and retain their primary functional and morphological properties. Therefore, THP-1 cells were used to study monocyte/macrophage function [28]. The CGF scaffold possesses tightly interwoven fibers that mimic the natural ECM, which ensures cell adherence and migration. Similar to mesenchymal cells [29], THP-1 macrophages can easily spread on the surface of CGF scaffolds, which indicates that the CGF scaffolds have good cytocompatibility with macrophages. In addition, the tough fibrin network of the CGF scaffold slowed the proteolysis of bioactive factors, which may modulate macrophage behavior.

CGF sustained the release of abundant growth factors such as platelet-derived growth factor (PDGF), endothelial growth factor (EGF), transforming growth factor and insulin-like growth factor, and various cytokines and chemokines such as IL-1β, IL-6 and IL-10, which are derived from platelets and leukocytes [2, 30]. These highly concentrated bioactive molecules should enable CGF to modulate the BPs of various cells, including monocytes and macrophages [16]. In the present study, CGF extract promoted THP-1 monocyte migration and monocyte/macrophage transition in the presence and absence of PMA treatment, which suggests that CGF initiates the immune response via promoting monocyte recruitment and maturation. We used low concentrations of CGF extract to mimic the sustained release feature *in vivo*, and the immunoregulatory effects of 20 and 50% CCM were not significantly different in most experiments. Consistent with our study, previous studies have shown that CGF extract modulated cell differentiation at relatively low concentrations (5–50%), and higher concentrations exerted no or negative effects [29, 31].

After monocytes mature into macrophages, macrophages further polarize into different phenotypes with distinct functional activities that are essential for tissue remodeling and healing [15]. The depletion of macrophages results in reduced cell recruitment, impaired angiogenesis and delayed tissue healing [32, 33]. Under complex physiological conditions, the macrophage population appears to be a mixture that can be dynamically shifted [16, 17]. The M1/M2 paradigm offers a simplified framework to evaluate the plastic transition of macrophage activities. Surface markers such as CD80 and CD86 are generally used to identify the M1 phenotype, and CD163 and CD206 are used as M2 macrophage markers [27]. We assessed the alterations in cell morphology and surface markers and found that CGF extract promoted THP-1 macrophage polarization toward the M2 subtype with upregulated CD163 expression. The biological role of PRP in macrophage polarization is controversial. One study found that PRP promoted M2 macrophage generation with increasing CD206 expression, but CD163 expression level was decreased [22]. Another study detected no clear effects [23]. Macrophages functionally secrete large quantities of diverse cytokines/chemokines, which makes these cells powerful modulators in the host immune system. CGF extract regulated the macrophage secretion of cytokines/chemokines related to migration, recruitment and adhesion, including the specific downregulation of IL-1β secretion and upregulation of RANTES expression. IL-1β has been proposed to be an initiator of infectious diseases and is regarded as a proinflammatory signal [34]. RANTES is a chemotactic cytokine that recruits leukocytes and lymphocytes to the injured region [35]. The chemotactic ability of CGF extract may be further enhanced by the increasing RANTES secretion. CGF extract promoted immune cell migration and recruitment while suppressing the release of inflammatory mediators. These functional effects might be attributed to a combination of the various growth factors and cytokines in CGF. Together, these results suggest that CGF regulates macrophage polarization and functional activities, which potentially restrict the inflammatory response and promote healing.

Macrophage polarization and function are highly plastic and dynamically shift depending on various signals and external cues. The highly CGFs and cytokines released from CGF are capable of regulating signaling dynamics, which define the cellular behavior of immune cells [5, 36, 37]. The diverse effects of the complex molecules in CGF might achieve a balance in the enriched JAK/STAT pathway based on the opposite effects observed on inflammatory cytokine and chemokine secretions. The present study identified the AKT pathway as the main signaling pathway through which CGF exerts its immunoregulatory effects. PRP also exerts a regenerative effect via the AKT pathway [38, 39], which supports our results. A variety of growth factors that are present in CGF extract can induce activation of AKT signaling. PDGF is a strong chemoattractant and growth factor that potently and specifically activates the AKT pathway in a PI3K-dependent manner [40]. Inulin signaling sustains and potentiates AKT pathway activation, and it interacts with the EGF network for signal propagation [41, 42]. The AKT pathway is a central node for the convergence of multiple signals to regulate macrophage survival, migration and polarization. AKT signaling activation is required for monocyte–macrophage homeostasis and M2 macrophage polarization [20, 43, 44]. Phosphorylated AKT is a negative regulator of inflammatory signals that restricts proinflammatory and excessive immune responses and promotes anti-inflammatory and repair activities in monocyte–macrophages [43, 45]. Activation of the AKT pathway also explains the aforementioned immunoregulatory effect of CGF on the macrophage-mediated immune response.

## Conclusions

Our study revealed the immunoregulatory role of CGF in macrophage functional activities *in vitro*. We characterized the morphological features and biocompatibility of CGF scaffolds. The abundance of bioactive factors in the CGF extract facilitated M2 macrophage polarization and modulated cytokine secretion, which were related to AKT signaling activation. These results shed light on the therapeutic potential and mechanisms of CGF in regulating the macrophage-mediated immune response, but *in vivo* experiments are needed to verify this result. Our data enrich the evidence that CGF is a promising regenerative biomaterial which is conducive to functional tissue regeneration.

## Supplementary data

Supplementary data are available at *REGBIO* online.

## Funding

This work was supported by the National Natural Science Foundation of China (81900989, 81870786), the Guangdong Basic and Applied Basic Research Foundation (2021A1515012475) and the China Postdoctoral Science Foundation (2020M672548).

*Conflict of interest statement.* None declared.

## Supplementary Material

rbab049_Supplementary_Data
