# Supplementary material for: Concentrated growth factor regulates the macrophage-mediated immune response
Source: Regen Biomater. 2021 Aug 17;8(6):rbab049. doi: 10.1093/rb/rbab049 (PMC8421811; doi:10.1093/rb/rbab049)
Supplement: rbab049_Supplementary_Data [file rbab049_supplementary_data.zip › Table S1.docx]

Figure 2:

|  |  | CTR | 10% CCM | 20% CCM | 50% CCM |
| --- | --- | --- | --- | --- | --- |
| THP-1 monocytes Transwell | n | 3 | 3 | 3 | 3 |
|  | Mean | 128.7 | 638.7 | 653.3 | 825 |
|  | Std. Deviation | 4.509 | 44.11 | 14.5 | 55.87 |
|  | Std. Error of Mean | 2.603 | 25.46 | 8.373 | 32.25 |
|  | Adjusted P Value |  | <0.0001  (10%CCM/CTR) | <0.0001  (20%CCM/CTR) | <0.0001  (50%CCM/CTR) |
| THP-1  monocytes Differentiation | n | 5 | 5 | 5 | 5 |
|  | Mean | 26.4 | 102 | 164.8 | 153.6 |
|  | Std. Deviation | 11.48 | 34.11 | 35.24 | 32.5 |
|  | Std. Error of Mean | 5.134 | 15.25 | 15.76 | 14.53 |
|  | Adjusted P Value |  | 0.0029  (10%CCM/CTR) | <0.0001  (20%CCM/CTR) | <0.0001  (50%CCM/CTR) |
| PMA induced-monocytes Differentiation | n | 5 | 5 | 5 | 5 |
|  | Mean | 178.4 | 653.4 | 781.2 | 537.4 |
|  | Std. Deviation | 68.79 | 139.1 | 161.8 | 98.7 |
|  | Std. Error of Mean | 30.76 | 62.23 | 72.36 | 44.14 |
|  | Adjusted P Value |  | <0.0001  (10%CCM/CTR) | <0.0001  (20%CCM/CTR) | 0.0008  (50%CCM/CTR) |

Figure 3C:

|  |  | CTR | 10% CCM | 20% CCM | 50% CCM |
| --- | --- | --- | --- | --- | --- |
| M1 macrophage (CD80+/CD86+) | n | 3 | 3 | 3 | 3 |
|  | Mean | 75.17 | 77.33 | 77.4 | 78.03 |
|  | Std. Deviation | 8.43 | 3.635 | 2.773 | 2.444 |
|  | Std. Error of Mean | 4.867 | 2.099 | 1.601 | 1.411 |
|  | Adjusted P Value |  | 0.9078  (10%CCM/CTR) | 0.9006  (20%CCM/CTR) | 0.8209  (50%CCM/CTR) |
| M2 macrophage (CD163+/  CD206+) | n | 3 | 3 | 3 | 3 |
|  | Mean | 11.73 | 13.27 | 19.5 | 23.13 |
|  | Std. Deviation | 1.041 | 0.4726 | 2.39 | 5.9 |
|  | Std. Error of Mean | 0.6009 | 0.2728 | 1.38 | 3.407 |
|  | Adjusted P Value |  | 0.8878  (10%CCM/CTR) | 0.0456  (20%CCM/CTR) | 0.0065  (50%CCM/CTR) |

Figure 3D:

|  |  | CTR | 10% CCM | 20% CCM | 50% CCM |
| --- | --- | --- | --- | --- | --- |
| CD163  MRNA EXPRESSION | n | 3 | 3 | 3 | 3 |
|  | Mean | 0.9963 | 2.291 | 3.225 | 5.185 |
|  | Std. Deviation | 0.1519 | 0.4557 | 0.5043 | 0.7207 |
|  | Std. Error of Mean | 0.08767 | 0.2631 | 0.2912 | 0.4161 |
|  | Adjusted P Value |  | 0.0329  (10%CCM/CTR) | 0.0016  (20%CCM/CTR) | <0.0001  (50%CCM/CTR) |
| CD80  MRNA EXPRESSION | n | 3 | 3 | 3 | 3 |
|  | Mean | 1.02 | 0.6667 | 0.5687 | 0.7943 |
|  | Std. Deviation | 0.1015 | 0.05859 | 0.03202 | 0.07488 |
|  | Std. Error of Mean | 0.05859 | 0.03383 | 0.01849 | 0.04323 |
|  | Adjusted P Value |  | 0.0008  (10%CCM/CTR) | 0.0002  (20%CCM/CTR) | 0.0120  (50%CCM/CTR) |

Figure 3E:

|  |  | CTR | 10% CCM | 20% CCM | 50% CCM |
| --- | --- | --- | --- | --- | --- |
| CD163  staining density | n | 3 | 3 | 3 | 3 |
|  | Mean | 20.7 | 28.6 | 30.1 | 35.6 |
|  | Std. Deviation | 0.882 | 4.02 | 2.31 | 3.08 |
|  | Std. Error of Mean | 0.509 | 2.32 | 1.33 | 1.78 |
|  | Adjusted P Value |  | 0.0219  (10%CCM/CTR) | 0.0089  (20%CCM/CTR) | 0.0005  (50%CCM/CTR) |

Figure 4E:

|  |  | CTR | 10% CCM | 20% CCM | 50% CCM |
| --- | --- | --- | --- | --- | --- |
| IL-1b  MRNA EXPRESSION | n | 3 | 3 | 3 | 3 |
|  | Mean | 1.047 | 0.8403 | 0.6624 | 0.5427 |
|  | Std. Deviation | 0.09082 | 0.05279 | 0.04012 | 0.05095 |
|  | Std. Error of Mean | 0.05244 | 0.03048 | 0.02317 | 0.02942 |
|  | Adjusted P Value |  | 0.0087  (10%CCM/CTR) | 0.0002  (20%CCM/CTR) | <0.0001  (50%CCM/CTR) |
| IL-7  MRNA EXPRESSION | n | 3 | 3 | 3 | 3 |
|  | Mean | 1.007 | 0.5553 | 0.257 | 0.3307 |
|  | Std. Deviation | 0.1428 | 0.1259 | 0.05429 | 0.05305 |
|  | Std. Error of Mean | 0.08247 | 0.0727 | 0.03134 | 0.03063 |
|  | Adjusted P Value |  | 0.0017  (10%CCM/CTR) | <0.0001  (20%CCM/CTR) | 0.0001  (50%CCM/CTR) |
| RANTES  MRNA EXPRESSION | n | 3 | 3 | 3 | 3 |
|  | Mean | 1.011 | 4.902 | 8.005 | 9.405 |
|  | Std. Deviation | 0.1833 | 0.1903 | 0.9195 | 0.9215 |
|  | Std. Error of Mean | 0.1058 | 0.1099 | 0.5309 | 0.532 |
|  | Adjusted P Value |  | 0.0003  (10%CCM/CTR) | <0.0001  (20%CCM/CTR) | <0.0001  (50%CCM/CTR) |
| MCP-1  MRNA EXPRESSION | n | 3 | 3 | 3 | 3 |
|  | Mean | 1.02 | 1.759 | 3.586 | 4.369 |
|  | Std. Deviation | 0.2365 | 0.6721 | 0.3433 | 1.422 |
|  | Std. Error of Mean | 0.1366 | 0.388 | 0.1982 | 0.8211 |
|  | Adjusted P Value |  | 0.5747  (10%CCM/CTR) | 0.0121  (20%CCM/CTR) | 0.0026  (50%CCM/CTR) |

Figure 4F:

|  |  | CTR | 10% CCM | 20% CCM | 50% CCM |
| --- | --- | --- | --- | --- | --- |
| IL-1β  protein expression | n | 3 | 3 | 3 | 3 |
|  | Mean | 6072 | 4382 | 3230 | 2120 |
|  | Std. Deviation | 150.3 | 40.23 | 64.33 | 63.34 |
|  | Std. Error of Mean | 86.77 | 23.23 | 37.14 | 36.57 |
|  | Adjusted P Value |  | <0.0001  (10%CCM/CTR) | <0.0001  (20%CCM/CTR) | <0.0001  (50%CCM/CTR) |
| RANTES protein expression | n | 3 | 3 | 3 | 3 |
|  | Mean | 25090 | 27397 | 31468 | 29506 |
|  | Std. Deviation | 353 | 977 | 468 | 567 |
|  | Std. Error of Mean | 204 | 564 | 270 | 327 |
|  | Adjusted P Value |  | 0.0056  (10%CCM/CTR) | <0.0001  (20%CCM/CTR) | <0.0001  (50%CCM/CTR) |

Figure 5A:

|  |  | CTR | 10% CCM | 20% CCM | 50% CCM |
| --- | --- | --- | --- | --- | --- |
| p-akt/akt | n | 3 | 3 | 3 | 3 |
|  | Mean | 0.4154 | 0.5734 | 2.732 | 1.575 |
|  | Std. Deviation | 0.02638 | 0.08742 | 0.2805 | 0.1925 |
|  | Std. Error of Mean | 0.01523 | 0.05047 | 0.162 | 0.1111 |
|  | Adjusted P Value |  | 0.5825  (10%CCM/CTR) | <0.0001  (20%CCM/CTR) | 0.0001  (50%CCM/CTR) |
| pi3k/gapdh | n | 3 | 3 | 3 | 3 |
|  | Mean | 1.012 | 1.295 | 1.177 | 1.125 |
|  | Std. Deviation | 0.1517 | 0.1346 | 0.09949 | 0.169 |
|  | Std. Error of Mean | 0.08757 | 0.07773 | 0.05744 | 0.09757 |
|  | Adjusted P Value |  | 0.0941 (10%CCM/CTR) | 0.3949 (20%CCM/CTR) | 0.6572 (50%CCM/CTR) |
| p-jak/jak | n | 3 | 3 | 3 | 3 |
|  | Mean | 0.6269 | 0.5104 | 0.4934 | 0.4414 |
|  | Std. Deviation | 0.09867 | 0.08326 | 0.1117 | 0.1247 |
|  | Std. Error of Mean | 0.05697 | 0.04807 | 0.06451 | 0.07201 |
|  | Adjusted P Value |  | 0.4380 (10%CCM/CTR) | 0.3413 (20%CCM/CTR) | 0.1473 (50%CCM/CTR) |
| p-stat3/  stat3 | n | 3 | 3 | 3 | 3 |
|  | Mean | 1.7 | 1.754 | 1.924 | 1.525 |
|  | Std. Deviation | 0.113 | 0.08488 | 0.1155 | 0.1886 |
|  | Std. Error of Mean | 0.06524 | 0.04901 | 0.06671 | 0.1089 |
|  | Adjusted P Value |  | 0.9191 (10%CCM/CTR) | 0.1602 (20%CCM/CTR) | 0.3051 (50%CCM/CTR) |

Figure 5B:

|  |  | CTR | 20% CCM | CCM+AKT inhibitor |
| --- | --- | --- | --- | --- |
| p-akt/akt | n | 3 | 3 | 3 |
|  | Mean | 0.6194 | 0.8403 | 0.6789 |
|  | Std. Deviation | 0.03024 | 0.05141 | 0.04579 |
|  | Std. Error of Mean | 0.01746 | 0.02968 | 0.02644 |
|  | Adjusted P Value |  | 0.0019 (20%CCM/CTR) | 0.0092 (CCM+AKTi/CCM) |
| pi3k/gapdh | n | 3 | 3 | 3 |
|  | Mean | 0.8517 | 1.035 | 0.9364 |
|  | Std. Deviation | 0.07938 | 0.1057 | 0.09653 |
|  | Std. Error of Mean | 0.04583 | 0.061 | 0.05573 |
|  | Adjusted P Value |  | 0.1199 (20%CCM/CTR) | 0.4574 (CCM+AKTi/CCM) |

Figure 5C:

|  |  | CTR | 20% CCM | CCM+AKT inhibitor |
| --- | --- | --- | --- | --- |
| PMA induced-monocytes Differentiation | n | 5 | 5 | 5 |
|  | Mean | 90 | 713.8 | 375.2 |
|  | Std. Deviation | 16.69 | 63.49 | 135.1 |
|  | Std. Error of Mean | 7.463 | 28.39 | 60.42 |
|  | Adjusted P Value |  | <0.0001  (20%CCM/CTR) | 0.0001  (CCM+AKTi/CCM) |

Figure 5E:

|  |  | CTR | 20% CCM | CCM+AKT inhibitor |
| --- | --- | --- | --- | --- |
| IL-1β  protein expression | n | 3 | 3 | 3 |
|  | Mean | 3916 | 3027 | 3810 |
|  | Std. Deviation | 275.4 | 44.81 | 153.6 |
|  | Std. Error of Mean | 159 | 25.87 | 88.66 |
|  | Adjusted P Value |  | 0.0025  (20%CCM/CTR) | 0.0048  (CCM+AKTi/CCM) |
| RANTES  protein expression | n | 3 | 3 | 3 |
|  | Mean | 7792 | 18784 | 12846 |
|  | Std. Deviation | 41.03 | 758.3 | 297.3 |
|  | Std. Error of Mean | 23.69 | 437.8 | 171.7 |
|  | Adjusted P Value |  | <0.0001  (20%CCM/CTR) | <0.0001  (CCM+AKTi/CCM) |

Figure 5F:

|  |  | CTR | 20% CCM | CCM+AKT inhibitor |
| --- | --- | --- | --- | --- |
| CD163  staining density | n | 3 | 3 | 3 |
|  | Mean | 19.6 | 31.3 | 22.4 |
|  | Std. Deviation | 3.23 | 6.14 | 6.1 |
|  | Std. Error of Mean | 1.32 | 2.51 | 2.49 |
|  | Adjusted P Value |  | 0.0046  (20%CCM/CTR) | 0.0272  (CCM+AKTi/CCM) |
