# Supplementary figures and images for: Concentrated growth factor regulates the macrophage-mediated immune response
Source: Regen Biomater. 2021 Aug 17;8(6):rbab049. doi: 10.1093/rb/rbab049 (PMC8421811; doi:10.1093/rb/rbab049)

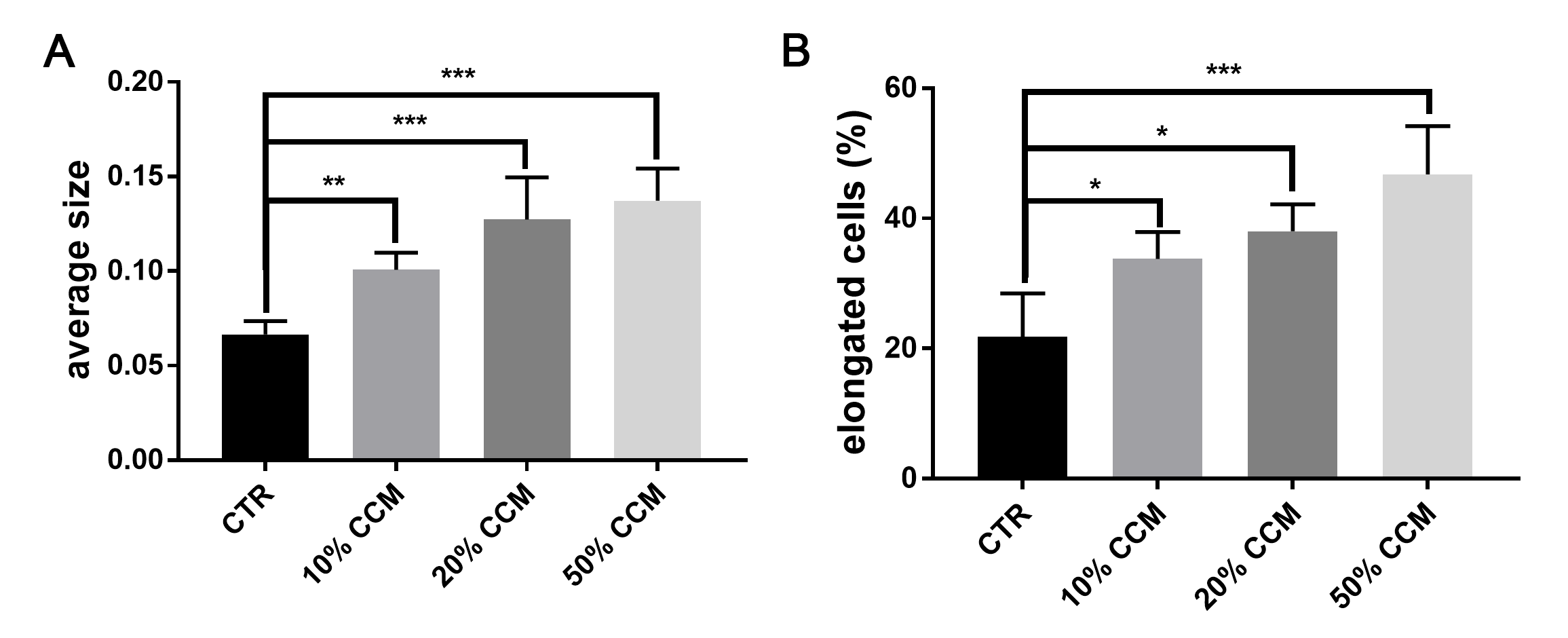

Supplement: rbab049_Supplementary_Data [file rbab049_supplementary_data.zip › Figure S1.tif]
